# Increases in Xu Zheng and Yu Zheng among Patients with Breast Cancer Receiving Different Anticancer Drug Therapies

**DOI:** 10.1155/2013/392024

**Published:** 2013-03-21

**Authors:** Sheng-Miauh Huang, Li-Yin Chien, Cheng-Jeng Tai, Ling-Ming Tseng, Ping-Ho Chen, Chen-Jei Tai

**Affiliations:** ^1^Department of Nursing, Mackay Medical College, New Taipei City 25245, Taiwan; ^2^Institute of Clinical and Community Health Nursing, National Yang-Ming University, Taipei 11221, Taiwan; ^3^Division of Hematology and Oncology, Department of Internal Medicine, Taipei Medical University Hospital, Taipei 11031, Taiwan; ^4^Department of Internal Medicine, School of Medicine, College of Medicine, Taipei Medical University, Taipei 11031, Taiwan; ^5^Division of General Surgery, Department of Surgery, Taipei Veterans General Hospital, Taipei 11217, Taiwan; ^6^Department of Surgery, National Yang-Ming University, Taipei 11221, Taiwan; ^7^Department of Traditional Chinese Medicine, Taipei Medical University Hospital, Taipei 11031, Taiwan; ^8^Department of OB/GYN, School of Medicine, College of Medicine, Taipei Medical University, Taipei 11031, Taiwan

## Abstract

*Aim*. The objectives of this study were to compare yang-xu, yin-xu, and yu among patients with breast cancer right before, one month after, and three months after receiving target, chemo, or combined therapy. *Method*. After recruiting 126 patients from 4 hospitals in northern Taiwan, a longitudinal study was carried out with 61 patients receiving chemotherapy, 30 receiving target therapy, and 35 receiving combined therapy. Yang-xu, yin-xu, and yu were assessed using the Traditional Chinese Medical Constitutional Scale (TCMCS), with higher scores indicating more xu and yu. *Results*. There were significant increases in yang-xu, yin-xu, and yu at 1 month and 3 months after than before the start of the chemotherapy, target, or combined therapy. Patients receiving combined therapy had significantly higher scores in yang-xu and yin-xu than patients receiving chemo or target therapy. A history of coronary heart disease was associated with more yin-xu. Those patients who had undergone a mastectomy were associated with less yu zheng than those patients who had not. *Conclusion and Implications*. TCM doctors should focus their treatment on dealing with xu and yu in order to support their patients, as they complete their modern anticancer treatments.

## 1. Introduction

Diagnostic and therapeutic systems in traditional Chinese medicine (TCM) are grounded in the identification of TCM zhengs, a concept that has been used for over 3,000 years. Zheng can be viewed as a syndrome and pattern that serves as the guide for TCM treatment [1–3]. One widely used system for Zheng classification was based on qi, blood, and fluid, which are viewed as the materials basis for living activities [4, 5]. Xu refers to a deficiency in qi and fluid, and yu refers to stasis or retention in blood [4]. Xu and yu suggest a state of imbalance and abnormal functioning of the organs in the human body [4, 5].

There are two types of xu: yang-xu and yin-xu. Yang-xu signifies a low energy (or qi) level in the physiological functioning of the body. Yin-xu is marked by insufficient yin fluid. Both yang-xu and yin-xu zhengs have been used to characterize the health status of the population [4–6]. Yu zheng usually occurs in diseased patients, because their qi and blood have been obstructed by the lesion site, which is prevalent among cancer patients [5, 6]. It is believed that a patient may suffer from different zhengs at the same time, and zheng is changing dynamically during the evolution of a disease and treatment course [7, 8].

Deaths due to breast carcinoma in 1990 and 2011 were 10.3 and 11.6 per 100,000 women in Taiwan. Although a trend toward an increased death rate from breast cancer has been reported in Taiwan, the rate was lower than the death rate in the United States (121.9 per 100,000 in 2008) [9, 10]. Diversified therapies have provided hope for extending disease-free survival among patients with breast cancer [11, 12]. Many patients with breast cancer, especially patients with HER2 overexpression, experience 3 sequential treatment stages, including chemotherapy, target plus chemotherapy, and lastly target therapy.

Past studies have shown that patients receiving taxane-based or anthracycline-based chemotherapies suffer severe physical symptoms, including musculoskeletal pain, fatigue, and nausea [13–15]. Outpatients receiving chemotherapy experienced on average 9.8, 14.4, and 13.7 symptoms at the start of chemotherapy, one week prior to the third cycle of chemotherapy, and one week prior to the fourth cycle of chemotherapy, respectively, showing a significant increase over time [13]. Other studies have reported that potential cardiac complications are frequent (more than 20%) and occur among patients taking trastuzumab [16, 17]. Previous studies of zhengs among cancer patients focused on molecular basis [18, 19]. No study has examined zhengs and the changes in zhengs among patients with breast cancer receiving different anticancer drug treatments.

**Figure 1:**
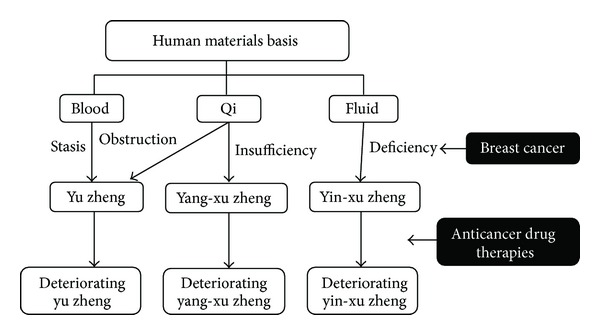
Proposed scheme for the change of xu and yu zheng among cancer patients receiving anticancer drug therapies.

The Taiwan National Health Insurance Program generally covers both modern medicine and traditional Chinese medicine (TCM) expenses in parallel. The prevalence of using complementary and alternative medicine among patients with cancer is high (19.5–85%) [20–24]. A significant number of patients with cancer concurrently receive modern anticancer treatments and traditional Chinese medicine (TCM) in Taiwan. Zheng classification served as a guide for TCM treatment. No study has evaluated the change in zheng among patients with cancer during the treatment period. The proposed scheme for the change of xu and yu zhengs among cancer patients receiving anticancer drug therapies is presented in Figure 1. The objectives of this study were to compare yang-xu, yin-xu, and yu among patients with breast cancer right before, one month after, and three months after receiving target, chemo, or combined therapy. In addition, demographic and disease factors related to yang-xu, yin-xu, and yu were examined.

## 2. Materials and Methods

This was an observational and longitudinal study. Women with breast cancer who were 18 years or older and who were about to start chemotherapy, target therapy plus chemotherapy, or target therapy were recruited from 4 teaching hospitals in northern Taiwan. Data were collected at pretreatment, 1 month, and 3 months after the treatment, using face-to-face interviews with structured questionnaires and medical chart reviews. Two research nurses who had undergone training for more than 6 months by a TCM doctor collected the data. The interviews were conducted either at the outpatient department or the ward at the patient's convenience. This study was approved by the human subjects committee at the hospitals (IRB approval numbers: CT-P-099007, 201006016IC, and TMUH-05-08-10).

### 2.1. Study Participants

Adult women with breast cancer who were about to start anticancer drug treatments during the period from June 2009 to July 2011 were recruited from 2 hospitals in Taipei City and 2 hospitals in Yilan county. Exclusion criteria were male, younger than 18 years of age, cancer recurrence, and unable to communicate in Mandarin Chinese or Taiwanese. Patients were ineligible if their breast cancer combined with other cancers. During the study period, 149 patients met the inclusion criteria. Twenty patients refused to participate at the first contact. Because one patient stopped the treatment and two patients refused further participation, we lost 3 patients during the study. Finally, 126 patients (84.0%) completed the study. Of the 126 patients, 61 patients received chemotherapy, 35 received target plus chemotherapy, and 30 received target therapy.

### 2.2. Treatment Protocol for the Chemo, Combined, and Target Therapies

 The treatment regimen followed the National Comprehensive Cancer Network guidelines. The patients in the target and combined groups had overexpression of the HER-2/neu oncogene, as confirmed by the pathology laboratory findings of immunohistochemistry (IHC) 3+ or positive fluorescence in situ hybridization (FISH). All patients in our study received the 3-week cyclic anthracycline-based, taxane-based plus trastuzumab, or trastuzumab therapy courses for at least 3 months. The chemotherapy group contained patients who received anthracycline-based intravenous chemotherapy consisting of either doxorubicin (60 mg/m^2^) or epirubicin (75 mg/m^2^) plus cyclophosphamide (600 mg/m^2^). Patients in this chemotherapy group had not previously received any chemotherapy. The combined-therapy group contained patients who were treated with taxane-based chemotherapy (a paclitaxel 175 mg/m^2^ IV or docetaxel 100 mg/m^2^ given intravenously) and trastuzumab (an initial loading dose of 8 mg/kg and 6 mg/kg thereafter, given intravenously). All patients in the combined-therapy group had been pretreated with anthracycline-based chemotherapy. The target therapy group contained patients who had been pretreated with chemotherapy and combined therapy and treated with continuous trastuzumab (6 mg/kg given intravenously).

### 2.3. Measurements

The study variables included demographics (age, marital status, work status, family income, and religion), clinical treatment/diagnostic characteristics (disease history, cancer stage, site of lesion, and type of mastectomy), use of TCM, and zhengs (yang-xu, yin-xu, and yu). The demographics and clinical treatment/diagnostic characteristics were collected before the start of treatment. Use of TCM was determined by simply asking patients whether they used TCM to treat their cancer at present.

The Traditional Chinese Medical Constitutional Scale (TCMCS) was the multisymptom patient-reported outcome measure for the zhengs. The TCMCS instrument was developed through literature review and a Delphi process by 26 experts [25, 26]. Reliability and validity of the scale were substantiated [25–28]. The TCMCS included 44 items covering 3 zheng identifications: yang-xu (19 items), yin-xu (19 items), and yu (16 items). Each item was rated on a 5-point Likert scale, ranging from 0 to 4. A higher score indicated more yang-xu, yin-xu, or yu zheng.

The yang-xu zheng implies that a person's energy (qi) to maintain body functions is insufficient resulting in symptoms such as fatigue, shortness of breath, chills, loose stool, and a large volume of urine. The yin-xu zheng means an insufficiency of body fluid and may present symptoms such as thirst, constipation, oral ulcers, and dry eye. The yu zheng features the imbalance and obstruction of qi and blood. People with the yu zheng may have painful lesions, numb limbs, sputum, or edema. The Cronbach's *α* for the 3 subscales was 0.83, 0.80, and 0.78, respectively. The TCMCS was collected at pretreatment, 1 month, and 3 months after the treatment began.

### 2.4. Data Analysis

Statistical analyses were performed using the Predictive Analytics Suite Workstation running version 18.0 software (PASW, IBM Corp., Somers, NY, USA). Individual variables were examined by percentages, means, and standard deviations. Differences among the 3 time points were examined by *χ* ^2^ statistics and ANOVA. Repeated measures ANOVA were used to examine the change of zheng at the 3 time points. As substantial individual differences were expected in the data, we used the generalized linear mixed model (GLMM) to perform multivariate analysis, examining factors associated with yang-xu, yin-xu, and yu [29]. The multivariate model would be adjusted for the significant variables by the bivariate analysis. In all analyses, a 5% significance level was used.

## 3. Results

### 3.1. Characteristics of the Participants

The mean age of the study women was 53.8 years (SD = 11.2). More than half of the women were married (63.5%) and unemployed (50.8%). Most patients had religious beliefs (86.5%) and sufficient family income (78.6%). Only seventeen patients (13.5%) did not undergo a mastectomy. The percentages at cancer stage I, II, III, and IV were 19.0%, 40.5%, 28.6%, and 11.9%, respectively. More patients in the combined therapy group had advanced stages of cancer (III and IV) than in the chemotherapy or target groups. Before treatment, there were no significant differences in TCM use among the chemotherapy, combined, and target therapy groups (Table 1). At 1 month and 3 months after the start of the treatment, patients in the chemotherapy group had a higher percentage of TCM use than those in the target and combined therapy groups (1 month after start of the treatment: *χ* ^2^ = 11.4, *P* < 0.01; 3 months after the start of treatment: *χ* ^2^ = 10.3, *P* < 0.01 (Table 1)).

### 3.2. Yang-Xu Zheng

**Figure 2:**
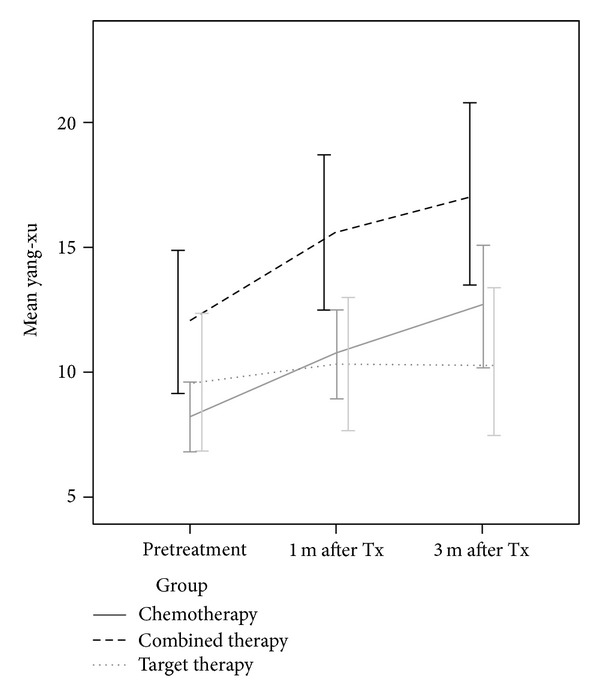
The change of yang-xu zheng with time.

Figure 2 displays the yang-xu scores at pretreatment, 1 month, and 3 months after the start of the treatment by treatment group (chemotherapy, combined, and target therapy). For patients receiving chemotherapy and combined therapy, the repeated measures ANOVA with post-hoc comparison showed that patients had worse yang-xu scores at 1 month or 3 months after the start of the treatment as compared to pretreatment (chemotherapy: 8.2 ± 5.4 at pretreatment, 10.7 ± 6.8 at 1 month after treatment began, and 12.6 ± 9.5 at 3 months after treatment began; *F* = 9.2, *P* < 0.01; combined therapy: 12.1 ± 8.3, 15.6 ± 8.9, and 17.1 ± 10.4; *F* = 5.7, *P* < 0.01). For patients receiving target therapy, the yang-xu score did not change significantly with the passage of time (9.6 ± 7.2, 10.4 ± 7.1, and 10.4 ± 7.6; *F* = 0.4, *P* = 0.69).

The patients receiving combined therapy had higher mean yang-xu scores than the patients receiving chemotherapy or target therapy (pretreatment: 12.1 ± 8.3 for the chemotherapy group, 8.2 ± 5.4 for the combined therapy group, and 9.6 ± 7.2 for the target therapy; *F* = 3.5, *P* = 0.03; at 1 month after treatment began: 10.7 ± 6.8, 15.6 ± 8.9, and 10.4 ± 7.1; *F* = 5.6, *P* < 0.01; at 3 months after treatment began: 12.6 ± 9.5, 17.1 ± 10.4, and 10.4 ± 7.6; *F* = 4.4, *P* = 0.01 (Figure 2)).

Bivariate analysis showed that age, diabetes, coronary heart disease, and mastectomy were significantly associated with the yang-xu score. The cancer stages and TCM differed significantly depending upon the therapy group. Therefore, we included and adjusted for those variables in the multivariate analysis. The GLMM model for the yang-xu score is presented in Table 2. Patients receiving combined therapy had significantly higher yang-xu scores as compared to the patients receiving chemotherapy (adjusted *β* = 3.88, 95% CI: 1.03–6.79) after we adjusted for the potential confounders. As compared to pretreatment, patients had higher yang-xu scores at 1 month and 3 months after the treatment began (1 month after treatment: adjusted *β* = 2.32, 95% CI: 1.02–3.61; 3 months after treatment: adjusted *β* = 3.65, 95% CI: 2.12–5.19 (Table 2)).

### 3.3. Yin-Xu Zheng

**Figure 3:**
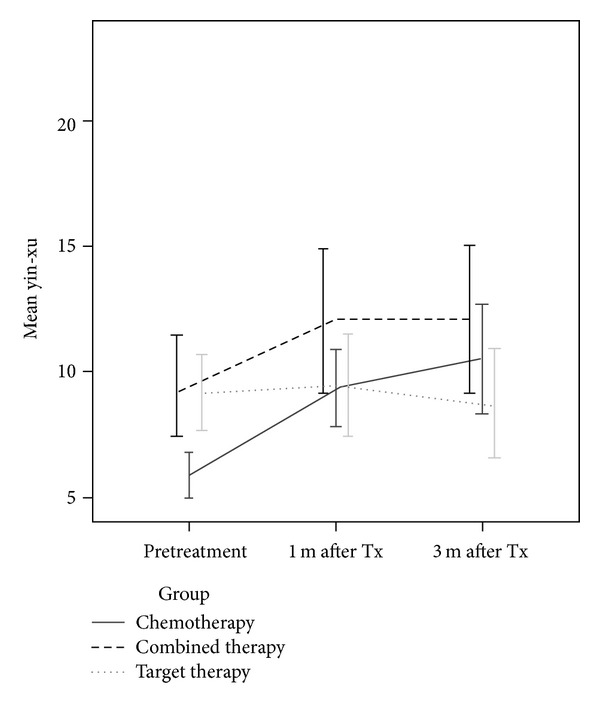
The change of yin-xu zheng with time.

Figure 3 displays the yin-xu scores at pretreatment, 1 month, and 3 months after the start of treatment by treatment group (chemotherapy, combined, and target therapy). For patients receiving chemotherapy and combined therapy, the repeated measures ANOVA with post-hoc comparison showed that patients had worse yin-xu scores at 1 month or 3 months after the start of the treatment as compared to pretreatment (chemotherapy: 5.9 ± 3.54 at pretreatment, 9.3 ± 5.7 at 1 month after treatment began, and 10.5 ± 8.3 at 3 months after treatment began; *F* = 13.2, *P* < 0.01; combined therapy: 9.4 ± 5.6, 12.0 ± 8.2, and 12.1 ± 8.3; *F* = 3.8, *P* = 0.03). The yin-xu scores for patients receiving target therapy did not change significantly at the later time points (9.1 ± 4.1, 9.4 ± 5.2, and 8.7 ± 5.7; *F* = 0.3, *P* = 0.72).

Before treatment, the patients receiving chemotherapy had lower mean yin-xu than those receiving combined or target therapy (5.9 ± 3.5 for the chemotherapy group, 9.4 ± 5.6 for the combined therapy group, and 9.1 ± 4.1 for target therapy; *F* = 9.7, *P* < 0.01 (Figure 3)). However, the differences were not statistically significant at 1 month and 3 months after treatment began.

Bivariate analysis showed that age, diabetes, and coronary heart disease were significantly associated with the yin-xu score. The cancer stage and TCM differed significantly depending on the therapy group. Therefore, we included and adjusted for those variables in the multivariate analysis. The GLMM model for the yin-xu score is presented in Table 2. Patients receiving combined therapy had significantly higher yin-xu scores as compared to the patients receiving chemotherapy (adjusted *β* = 2.55, 95% CI: 0.41–4.68), after adjusting for the given potential confounders. As compared to pretreatment, the patients had higher yin-xu scores at 1 month and 3 months after the treatment began (1 month after treatment: adjusted *β* = 2.45, 95% CI: 1.38–3.52; 3 months after treatment: adjusted *β* = 2.83, 95% CI: 1.51–4.14 (Table 2)). Patients with coronary heart disease had higher yin-xu scores (adjusted *β* = 5.07, 95% CI: 1.95–8.19 (Table 2)).

### 3.4. Yu Zheng

**Figure 4:**
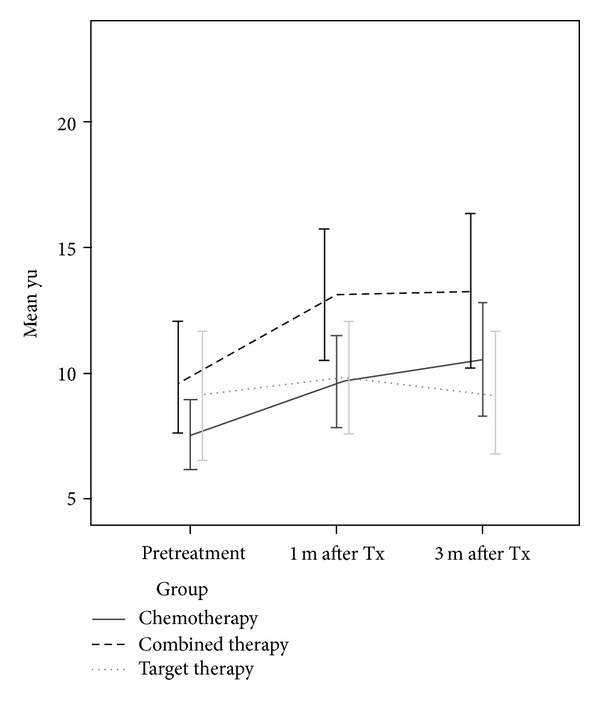
The change of yu zheng with time.

Figure 4 displays the yu-zheng scores at pretreatment, 1 month, and 3 months after the start of treatment by treatment group (chemotherapy, combined, and target therapy). For patients receiving chemotherapy and combined therapy, the repeated measures ANOVA with post-hoc comparison showed that patients had worse yu-zheng scores at 1 month or 3 months after the start of the treatment as compared to pretreatment (chemotherapy: 7.6 ± 5.1 at pretreatment, 9.7 ± 6.9 at 1 month after treatment began, and 10.5 ± 8.8 at 3 month after treatment began; *F* = 4.4, *P* = 0.01; combined therapy: 9.9 ± 6.3, 13.1 ± 7.5, and 13.3 ± 8.8; *F* = 5.7, *P* < 0.01). The yu-zheng scores for patients receiving target therapy did not change significantly as time passed (9.1 ± 6.8, 9.8 ± 5.9, and 9.2 ± 6.4; *F* = 0.4, *P* = 0.69).

Patients receiving combined therapy seemed to have higher mean yu-zheng scores than those receiving chemotherapy or target therapy, but the differences were not statistically significant (Figure 4). Bivariate analysis showed that age, diabetes, coronary heart disease, and mastectomy were significantly associated with the yu-zheng scores. The cancer stage and TCM differed significantly depending on the therapy group. Therefore, we included and adjusted for those variables in the multivariate analysis. The GLMM model for the yu-zheng score is presented in Table 2. There were no significant differences in the yu-zheng scores among the three groups after adjusting for the given potential confounders. As compared to pretreatment, patients had higher yu scores at 1 month and 3 months after the treatment began (1 month after treatment: adjusted *β* = 2.05, 95% CI: 0.86–3.24; 3 months after treatment: adjusted *β* = 2.36, 95% CI: 1.03–3.69 (Table 2)). Patients with mastectomies had lower yu-zheng scores (adjusted *β* = −3.38, 95% CI: −6.51 to −0.25).

## 4. Discussion

Our study found that the yang-xu, yin-xu, and yu-zhengs were worse among the patients with breast cancer after therapy. This implies that treatment with oncologic drugs, whether anthracycline-based, taxane-based plus trastuzumab, or trastuzumab therapy, deteriorates the whole body over time. In practice, the patients in the trastuzumab therapy group would undergo treatment for more than one year. We need to develop a care plan for these patients in long-term therapy in a future study. For example, the classic TCM herbal formula, Shi Quan Da Bu Tang (SQDBT, also known as Juzen-taiho-to or TJ-48) is a tonic formula, which could be used to deal with Xu. We previously reported that SQDBT is effective in alleviating hematotoxicity among patients with breast cancer receiving chemotherapy [30]. Nonetheless, more study is needed in order to examine the effectiveness of TCM treatment based on Zheng classification among patients with cancer.

Our study showed that the patients receiving combined therapy had significantly worse scores in yang-xu and yin-xu zhengs than the patients receiving chemotherapy or target therapy. The patients receiving combined therapy also had worse scores in yu zheng, though the difference was not statistically significant. It seemed that the more anticancer drugs the patients used, the worse xu zheng score the patients reported. Nonetheless, TCM doctors should pay more attention to patients receiving combined therapy, with special emphasis on dealing with their xu zheng.

Based on the bivariate analysis, we found that the yang-xu, yin-xu, and yu of the patients receiving target therapy did not change significantly at 1 month or 3 months after the treatment relative to before the treatment. However, the multivariate analysis results showed a worsening in yang-xu, yin-xu, and yu after the treatment than before, even among patients receiving target therapy. The differences in the results could be due to the small number of patients receiving target therapy in the study. Since the patients receiving target drugs will continue taking the drug for more than 1 year, the patients need to be followed for a longer period of time in order to better examine its effect.

A previous study using national data reported that the rate of TCM use among patients with liver cancer was about 20% [24]. We found that 21% of the patients with breast cancer used TCM before starting their anticancer drug treatment. As compared to before the start of the treatment, more patients used TCM in the chemotherapy group, but fewer patients in the combined or target group. In addition, the rate of TCM use at 1 month and 3 months after the start of drug treatment was the highest among the chemotherapy group, followed by the combined therapy group, and lastly the target group. Those differences have not been reported by other previous studies.

A future study with a larger sample size is needed to validate our findings. In our study, TCM use was not significantly related to zheng changes, which could be due to the small number of patients using TCM. In addition, TCM users tended to have more xu and yu than nonusers before the treatment (data not shown). Since duration of TCM use, contents of TCM prescription, and patients' actual intake of TCM drugs were not available in the study, and subsequent studies need to incorporate those information and examine the effectiveness of TCM treatment on decreasing xu and yu among patients with cancer receiving anticancer drug treatments.

Coronary heart disease was found to be associated with increased yin-xu among the study participants. Our study participants received anticancer drugs, which may induce chronic cardiotoxicity, heart failure, and hence more yin-xu [31]. For example, the anthracycline-based drugs or trastuzumab may exacerbate the heart problems among patients with coronary heart disease. The yin-xu zheng manifests itself as thirst, constipation, oral ulcers, or dry eye. Those symptoms could be associated with drugs for heart diseases such as diuretics.

We also found that patients who had undergone mastectomies had more yu than those who had not. Larger tumor size could be the reason why the patients did not receive a mastectomy. Their larger tumor lesions may have interfered with the movement of qi and blood, and thus more yu [25]. TCM doctors should be aware of the potential effects of coronary heart disease on yin-xu and of mastectomy on yu in order to provide appropriate treatment for those patients.

We used the TCMCS to measure xu and yu in the study. Since the TCMCS is a structured questionnaire, it could be easily used by TCM doctors or other health professionals with only basic trainings. Our study demonstrated that the TCMCS could be used prospectively to document the change of xu and yu zhengs among cancer patients. Nonetheless, whether TCM doctors could incorporate the TCMCS in their clinical practice to monitor status of individual patients which merits further study. The interviewers did not blind to the treatment groups of the patients in the study. Since all the participants were under anticancer drug treatment, this may not have influenced validity of the results.

## 5. Conclusion

The yang-xu, yin-xu, and yu zhengs among the patients still deteriorated during chemotherapy and combined therapy. Underlying disease and treatment were strong factors predicting the changes to the different zhengs. Based on the changes in those zhengs, TCM doctors should develop standardized and scientific approaches to preventing and alleviating complications. The improvement and control of those zhengs would support breast cancer patients in completing modern anticancer treatments.

## Figures and Tables

**Table 1:** Characteristics of the study participants by the chemotherapy, combined, and target therapy groups (*N* = 126).

	Therapy group			*F*/*χ* ^2^	*P*
	Chemotherapy (*n* = 61) *n* (%)	Combined therapy (*n* = 35) *n* (%)	Target therapy (*n* = 30) *n* (%)		
Age (mean ± SD)	52.6 ± 11.2	56.5 ± 12.1	53.0 ± 9.9	1.5	0.23
Marital status				2.3	0.90
Single	11 (18.0)	8 (22.9)	4 (13.3)		
Married	40 (65.6)	19 (54.3)	21 (70)		
Widowed	7 (11.5)	5 (14.3)	3 (10)		
Divorced	3 (4.9)	3 (8.6)	2 (6.7)		
Currently working				1.7	0.44
No	29 (47.5)	21 (60)	14 (46.7)		
Yes	32 (52.5)	14 (40)	16 (53.3)		
Family Income					
Insufficient	16 (26.2)	6 (17.1)	5 (16.7)		
Sufficient	45 (73.8)	29 (82.9)	25 (83.3)		
Religion				1.8	0.41
No	7 (11.5)	7 (20)	3 (10)		
Yes	54 (88.5)	28 (80)	27 (90)		
Diabetes				1.2	0.54
No	56 (91.8)	33 (94.3)	26 (86.7)		
Yes	5 (8.2)	2 (5.7)	4 (13.3)		
Hypertension				1.0	0.62
No	49 (80.3)	29 (82.9)	22 (73.3)		
Yes	12 (19.7)	6 (17.1)	8 (26.7)		
Coronary heart disease				0.4	0.80
No	55 (90.2)	31 (88.6)	28 (93.3)		
Yes	6 (9.8)	4 (11.4)	2 (6.7)		
Cancer stage				10.5	<0.01
I and II	43 (70.5)	13 (37.1)	19 (63.3)		
III and IV	18 (29.5)	22 (62.9)	11 (36.7)		
Lesion				3.7	0.45
Left	29 (47.5)	18 (51.4)	13 (43.3)		
Right	30 (49.2)	15 (42.9)	13 (43.3)		
Bilateral	2 (3.3)	2 (5.7)	4 (13.3)		
Mastectomy				6.6	0.16
No	10 (16.4)	7 (20)	0 (0)		
Partial	38 (62.3)	20 (57.1)	21 (70)		
Total	13 (21.3)	8 (22.9)	9 (30)		
Use of TCM					
Before treatment				1.7	0.43
No	49 (79.0)	28 (82.4)	27 (90)		
Yes	13 (21.0)	6 (17.6)	3 (10)		
1 month after the start of treatment				11.4	<0.01
No	43 (69.4)	30 (88.2)	29 (96.7)		
Yes	19 (30.6)	4 (11.8)	1 (3.3)		
3 month after the start of treatment				10.3	<0.01
No	42 (68.9)	30 (85.7)	28 (93.3)		
Yes	20 (31.1)	4 (14.3)	2 (6.7)		

*F* and χ^2^ are the respective statistical values from the one-way ANOVA and chi-squared tests; the significance level was set at *P* < 0.05.

**Table 2:** The GLMM results on the yang-xu, yin-xu, and yu zhengs.

	Yang-xu zheng			Yin xu zheng			Yu zheng		
	Estimate	SE	*P *	Estimate	SE	*P *	Estimate	SE	*P *
Intercept	8.39	3.28	0.01	6.64	2.26	<0.01	9.84	2.85	<0.01
Target therapy stage^a^	0.33	1.50	0.83	0.89	1.10	0.42	0.81	1.31	0.54
Combined therapy stage^a^	3.88	1.44	0.01	2.55	1.08	0.02	2.40	1.25	0.06
3 months after treatment^b^	3.65	0.78	<0.01	2.83	0.67	<0.01	2.36	0.67	<0.01
1 month after treatment^b^	2.32	0.66	<0.01	2.45	0.54	<0.01	2.05	0.60	<0.01
Age	0.03	0.06	0.60	−0.02	0.04	0.69	0.00	0.05	0.95
Diabetes	1.95	2.17	0.37	1.08	1.62	0.51	2.37	1.88	0.21
Coronary heart disease	3.35	2.14	0.12	5.07	1.58	<0.01	3.37	1.86	0.07
Cancer stage III or IV	0.51	1.28	0.69	0.46	0.92	0.62	0.61	1.11	0.59
TCM Treatment^c^	1.93	1.13	0.09	0.95	0.90	0.29	1.17	1.00	0.24
Mastectomy^d^	−2.89	1.82	0.11				−3.38	1.58	0.04

^a^Anthracycline-based chemotherapy, ^b^pretreatment, ^c^without TCM treatment, and ^d^without mastectomy.

GLMM: generalized linear mixed model, AR(1). The significance level was set at *P* < 0.05.
